# Spectral gene set enrichment (SGSE)

**DOI:** 10.1186/s12859-015-0490-7

**Published:** 2015-03-03

**Authors:** H Robert Frost, Zhigang Li, Jason H Moore

**Affiliations:** 1 Institute of Quantitative Biomedical Sciences, Geisel School of Medicine, Lebanon, NH, 03756 USA; 2 Section of Biostatistics and Epidemiology, Department of Community and Family Medicine, Geisel School of Medicine, Lebanon, NH, 03756 USA; 3 Department of Genetics, Dartmouth College, Hanover, NH, 03755 USA

## Abstract

**Background:**

Gene set testing is typically performed in a supervised context to quantify the association between groups of genes and a clinical phenotype. In many cases, however, a gene set-based interpretation of genomic data is desired in the absence of a phenotype variable. Although methods exist for unsupervised gene set testing, they predominantly compute enrichment relative to clusters of the genomic variables with performance strongly dependent on the clustering algorithm and number of clusters.

**Results:**

We propose a novel method, spectral gene set enrichment (SGSE), for unsupervised competitive testing of the association between gene sets and empirical data sources. SGSE first computes the statistical association between gene sets and principal components (PCs) using our principal component gene set enrichment (PCGSE) method. The overall statistical association between each gene set and the spectral structure of the data is then computed by combining the PC-level p-values using the weighted Z-method with weights set to the PC variance scaled by Tracy-Widom test p-values. Using simulated data, we show that the SGSE algorithm can accurately recover spectral features from noisy data. To illustrate the utility of our method on real data, we demonstrate the superior performance of the SGSE method relative to standard cluster-based techniques for testing the association between MSigDB gene sets and the variance structure of microarray gene expression data.

**Conclusions:**

Unsupervised gene set testing can provide important information about the biological signal held in high-dimensional genomic data sets. Because it uses the association between gene sets and samples PCs to generate a measure of unsupervised enrichment, the SGSE method is independent of cluster or network creation algorithms and, most importantly, is able to utilize the statistical significance of PC eigenvalues to ignore elements of the data most likely to represent noise.

**Electronic supplementary material:**

The online version of this article (doi:10.1186/s12859-015-0490-7) contains supplementary material, which is available to authorized users.

## Background

Gene set testing has become an indispensable tool for the analysis and interpretation of high dimensional genomic data, including measures of DNA sequence variation, DNA methylation, RNA expression and protein abundance [1,2]. By focusing on the collective effect of biologically meaningful groups of genomic variables, rather than just the marginal effect of individual variables, gene set testing methods can significantly improve statistical power, replication of results and biological interpretation. Because of these benefits, significant effort has been devoted over the last decade to building large repositories of functional gene sets [3-5], creating methods for refining and customizing these gene set collections [6-8] and developing effective statistical techniques for gene set testing [9-13].

Gene set testing is normally used to quantify the association between functional groups of genomic variables and a clinical phenotype, e.g., cancer case/control status. Many important use cases exist, however, where a gene set-based interpretation of genomic data is desired in the absence of a phenotype variable, e.g., case-only data collections. For such unsupervised applications, the standard approach for gene set testing involves the computation of the association between gene sets and a categorical variable defined by disjoint clusters of genomic variables. Such methods typically compute the association between each gene set and the variable clustering using either information theoretic measures [14,15] or contingency table-based statistical tests which incorrectly assume independence among the genomic variables [16-18]. Although these techniques provide a measure of gene set enrichment for a given clustering of genomic data, the motivation for most methods is cluster evaluation rather than unsupervised biological interpretation. Cluster-based gene set enrichment results are strongly dependent on the clustering method employed and the number of computed clusters. This sensitivity to the clustering method and number of clusters makes these methods very useful for clustering evaluation but unreliable as general measures of unsupervised gene set enrichment. Specifically, since these methods advocate the use gene set enrichment results to select the clustering method and number of clusters, instead of often unreliable metrics such as the gap statistic [19] or average silhouette width [20], it is unclear what clustering method or number of clusters should be used if the goal is unbiased gene set testing.

An alternative approach for unsupervised gene set testing with many similarities to cluster-based methods is gene set enrichment of gene networks. This approach typically involves the computation of a network from a genomic dataset with network nodes represented by genomic variables, e.g., a co-expression network for gene expression data [21], a community detection algorithm is then used to decompose the network nodes into distinct groups and, finally, gene set testing is performed relative to each community or all communities. If the network communities are treated like gene clusters, the same information theoretic or contingency table-based methods employed for cluster-based enrichment can be used to calculate the association between gene sets and network communities. Approaches have also been developed that directly leverage the network structure to test for the association between gene sets and single network nodes [22] or groups of nodes [23]. Similar to cluster-based approaches, gene set enrichment of networks is highly dependent on the method used to build the network from genomic data and algorithms employed for community detection.

Methods have also been developed to test the association between gene sets and latent variables computed from genomic data sets via techniques such as principal component analysis (PCA) or independent component analysis (ICA). Most of these methods test for the association with just a single latent variable and employ an anti-conservative contingency-table based test on a dichotomized version of the loadings for the latent variable [24-26]. An exception is our recently developed principal component gene set enrichment (PCGSE) method [27] that performs competitive gene set testing relative to each PC using a statistical test that adjusts for correlation among gene set members. Similar to single cluster gene set testing methods, methods that perform gene set testing relative to a single latent variable can only provide an interpretation for a portion of a genomic data set. To test for the association between gene sets and a collection of latent variables representative of the entire data set, matrix correlation methods [28,29] have been employed, however, such methods are dependent on the number of latent variables included in the test and can only be used for self-contained gene set testing [30] (*Q*_2_ in the terminology of Tian et al. [31]).

Effective methods do not currently exist for unsupervised gene set testing against a competitive null hypothesis that are independent of specific cluster analysis or network analysis approaches. To address this shortcoming, we have developed spectral gene set enrichment (SGSE), an approach for unsupervised competitive testing of the association between gene sets and empirical data sources independent of cluster or network analysis. The SGSE method first computes the statistical association between gene sets and principal components (PCs) using our principal component gene set enrichment (PCGSE) method. The overall statistical association between each gene set and the spectral structure of the data is then computed by combining the PC-level p-values using the weighted Z-method with weights set to the PC variance scaled by lower-tailed p-value computed for the PC variance according to the Tracy-Widom distribution. Although described in the context of gene sets and genomic data, the SGSE method can be used to compute the statistical association between any collection of variable groups and the spectral structure of any empirical data set. To facilitate use of the SGSE method by other researchers, we have included an implementation of the algorithm in the PCGSE R package, which is available from the CRAN repository. Using simulated gene expression data and simulated gene sets, we show that the SGSE method can accurately recover known spectral features from noisy data, features that are undetectable using cluster-based approaches. To illustrate the utility of our method on real genomic data, we compare the performance of the SGSE method and a cluster-based technique on testing the association between MSigDB gene sets and the spectra of two cancer microarray gene expression data sets.

## Methods

### SGSE inputs

Similar to our principal component gene set enrichment (PCGSE) method [27], the SGSE method takes as input both an *n*×*p* genomic data matrix **X** quantifying *p* genomic variables under *n* experimental conditions and an *f*×*p* binary annotation matrix **A** that specifies the association between the *p* genomic variables and *f* functional categories.

The genomic data held in **X**, e.g., mRNA expression levels, will be modeled as a sample of *n* independent observations from a *p*-dimensional random vector **x**. It is assumed that any desired transformations on **X** have been performed and that missing values have been imputed or removed. Although the SGSE method is robust to departures from multivariate normality, as discussed in Section “PC statistical significance” below, it will be assumed that **x**∼*M* *V* *N*(***μ***,***Σ***) with correlation matrix **P**. This distributional assumption is usually well justified since sources of genomic data, especially gene expression data, are typically well approximated by a multivariate normal distribution after appropriate transformations.

The rows of **A** represent *f* biological categories, e.g., KEGG pathways or GO categories, and the elements *a*_*i*,*j*_ hold indicator variables whose value depends on whether an annotation exists between the function *i* and genomic variable *j*.

### SGSE algorithm

Enrichment of the gene sets defined by **A** relative to the spectra of **X** is performed using the following steps, which are explained in detail in sections “PCA for SGSE” thru “Combined significance of PCGSE p-values” below.

1. Perform PCA on **X**.
2. Determine *q*, the number of PCs used to represent the spectra of **X**.
3. For all *q* PCs, use the PCGSE method to compute the statistical significance of the association between each PC and each of the *f* gene sets defined by **A** according to a competitive null hypothesis.
4. Compute the statistical significance of the association between each of the *f* gene sets and the spectra of **X** using the weighted Z-method on the *q* PCGSE p-values with weights based on the PC variances optionally scaled according to PC statistical significance.

### PCA for SGSE

Because PCs are not invariant under scaling of the data [28], the PCA solution for SGSE is computed on a mean centered and standardized version of $\mathbf {X}, \tilde {\mathbf {X}}$. The PC loading vectors and variances of $\tilde {\mathbf {X}}$ are thus the eigenvectors and eigenvalues of:

(1) $$ \mathbf{S} = \frac{1}{n-1} \tilde{\mathbf{X}}^{T}\tilde{\mathbf{X}}  $$

The spectral decomposition of **S** is defined as:

(2) $$  \mathbf{S} = \sum\limits_{i=1}^{r_{\tilde{\mathbf{X}}}} \lambda_{i} v_{i} {v_{i}^{T}}  $$

where $r_{\tilde {\mathbf {X}}}$ is the rank of $\tilde {\mathbf {X}}, \lambda _{i}$ is the *i* ^*th*^ eigenvalue of **S** and the variance of the *i* ^*th*^ PC of $\tilde {\mathbf {X}}, v_{i}$ is the *i* ^*th*^ eigenvector of **S** and the loadings for the *i* ^*th*^ PC of $\tilde {\mathbf {X}}$ and $\tilde {\mathbf {X}} v_{i}$ is the *i* ^*th*^ PC. It is assumed that the eigenvalues are sorted in decreasing order: $\lambda _{1} \geq \lambda _{2} \geq \ldots \geq \lambda _{r_{\tilde {\mathbf {X}}}}$. Because **x**∼ MVN, (*n*−1)**S** is approximately Wishart distributed:

(3) $$ (n-1)\mathbf{S} = \tilde{\mathbf{X}}^{\mathbf{T}}\tilde{\mathbf{X}} \sim W(n, \mathbf{P})  $$

Similar to PCGSE, the PCA solution for SGSE is realized via the singular value decomposition (SVD) of a, $\tilde {\mathbf {X}} = \mathbf {U} \mathbf {E} \mathbf {V}^{T}$, where the columns of **V** represent the PC loading vectors, the entries in the diagonal matrix **E** are proportional to the square roots of the PC variances and the columns of **U** **E** are the PCs.

### PC statistical significance

Random matrix theory (RMT) methods provide useful distributional results for the bulk and extreme eigennvalues of matrices with Wishart distributions [32,33]. As outlined by Johnstone [32], the principal eigenvalue of a sample covariance matrix with a *white* Wishart distribution, where *white* implies that **Σ**=**I**, tends to a distribution described by a *Tracy-Widom* law of order 1 [34]. Specifically, if *n*,*p*→*∞*,*n*/*p*→*η*≥1, then the distribution of the rescaled principal eigenvalue:

(4) $$  \frac{\lambda_{1} - \mu(\,p, n)}{\sigma(\,p, n)}  $$

tends to a *Tracy-Widom* law of order 1, where $\mu (p, n) = \frac {(\sqrt {n-1} + \sqrt {p})^{2}}{n}$ and $\sigma (\,p, n) = \frac {\sqrt {n-1} + \sqrt {p}}{n} \left (\frac {1}{\sqrt {n-1}} +\frac {1}{\sqrt {p}} \right)^{1/3}$.

For *p*>*n*, the *Tracy-Widom* distribution still holds with *p* and *n* simply reversed in the *μ*(*p*,*n*) and *σ*(*p*,*n*) parameter definitions. Although an asymptotic result, this distribution was found to hold well even for *p* and *n* values as small as *p*=20 and *n*=5 [32]. It also holds well even when the underlying distribution of the elements of $\tilde {\mathbf {X}}$ is not normal [35].

In cases where **Σ** has *q* variances greater than 1, i.e., a spiked covariance model, Johnstone [32] demonstrated that this distribution approximates the distribution of the (*q*+1)^*th*^ eigenvalue but with slightly heavier tails. This result can therefore be used to compute a conservative statistical significance for all of the PCs of $\tilde {\mathbf {X}}$ based on the associated eigenvalues under a null hypothesis of uncorrelated MVN data (e.g., use of PCA for population genetics [36]). Specifically, the statistical significance of PC *i* can be determined by first computing a *Tracy-Widom* distributed statistic, *t* *w*_*i*_, for the eigenvalue, *λ*_*i*_, associated with the PC using equation 4:

(5) $$  {tw}_{i} = \frac{\lambda_{i} - \mu(p-i+1, n)}{\sigma(p-i+1, n)}  $$

Under the *H*_0_ that the data is a sample from a MVN distribution with no pair-wise correlation among the individual variables, the p-value for PC *i* is then computed as the probability of a *Tracy-Widom* law of order 1 statistic more extreme than *t* *w*_*i*_:

(6) $$  \text{p-value}_{\text{PC}_{i}} = 1-F_{\text{TW}}({tw}_{i})  $$

where *F*_*TW*_() is the cumulative distribution function of a *Tracy-Widom* law of order 1 random variable. This probability can be computed using either numerical lookup tables such as those supported by the RMTstat R package or via the Gamma approximation to the *Tracy-Widom* distribution detailed in Chiani [37]. The SGSE method currently uses the Gamma approximation for more accurate coverage of the tails of the distribution.

### Number of PCs used to represent data

The SGSE method supports three options for determining *q*, the number of PCs used to represent the spectra of **X**:

1. All PCs with non-zero variance: $q = \max _{i} \lambda _{i} > 0 = r_{\tilde {\mathbf {X}}}$
2. All statistically significant PCs at a specific *α* level where statistical significance of a given PC *i* is determined according (6).
3. A specified number, *q* ^∗^, with the constraint that *q* ^∗^ cannot be greater than the number of PCs with non-zero variance: $q = q^{*}, \text {s.t.} ~q^{*} \leq r_{\tilde {\mathbf {X}}}$. If specified, *q* ^∗^ will typically be set to a small number, e.g., 1 or 2, to minimize computational cost of the SGSE algorithm.

### PCGSE for SGSE

Our PCGSE method [27] is used to compute the statistical significance of the association between each of the *f* gene sets defined in **A** and each of the first *q* PCs of $\tilde {\mathbf {X}}$ where *q* is determined using one of the three methods detailed in Section “Number of PCs used to represent data” above. Let the p-value computed via PCGSE for PC *i* and gene set *j* be represented using the notation $\phantom {\dot {i}\!}\text {p-value}_{\text {PC}_{i}, \text {gs}_{j}}$.

Although any supported PCGSE options can be used with SGSE, by default, the SGSE method executes PCGSE using the Fisher-transformed Pearson correlation coefficient between each variable and each PC as the gene-level test statistic and the correlation-adjusted standardized mean difference statistic as the gene set test statistic with statistical significance of the gene set test statistic under a competitive *H*_0_ computed using a two-sided t-test as detailed in Frost et al. [27].

### Combined significance of PCGSE p-values

For each of the *f* gene sets, the p-values computed via PCGSE for the *q* selected PCs of $\tilde {\mathbf {X}}$ are combined using the weighted Z-method, a generalization of the untransformed Z-transform test [38,39]. The weighted Z-method combines Z-statistics generated for each of multiple independent p-values using weights specific to each p-value. This approach for combining p-values is justified for SGSE under the assumption of multivariate normality for **x** making both the uncorrelated PCs of $\tilde {\mathbf {X}}$, and the p-values generated by PCGSE with respect to those PCs, independent. If **x** is significantly non-Gaussian, then the PC-specific p-values will be dependent and techniques such as Kost’s method [40] or a generalized version of Fisher’s method [41] must be employed instead of the weighted Z-method. In the context of SGSE, a weighed Z-statistic is generated for each of the *f* gene sets as follows:

(7) $$  Z_{\text{gs}_{j}} = \frac{\sum_{i=1}^{q} w_{i} \Phi^{-1} \left(1-\text{p-value}_{\text{PC}_{i}, \text{gs}_{j}}\right)}{\sqrt{\sum_{i=1}^{q} {w_{i}^{2}} }}  $$

where *w*_*i*_ is a weight specific to PC *i* of $\tilde {\mathbf {X}}$ and *Φ* ^−1^() is the inverse standard normal CDF. Two options are supported for determining the PC-specific weights, *w*_*i*_:

1. The weight is set to the variance of each PC: *w*_*i*_=*λ*_*i*_
2. The weight is set to the variance of each PC scaled by the lower-tailed p-value computed for the PC variance according to the *Tracy-Widom* distribution as detailed in Section “PC statistical significance”: $\phantom {\dot {i}\!} w_{i} = (1-\text {p-value}_{\text {PC}_{i}}) \lambda _{i} = F_{\text {TW}}(tw_{i}) \lambda _{i}$

The overall p-value representing the statistical significance of the association between gene set *j* and the spectra of **X** is then computed using a one-sided z-test on $\phantom {\dot {i}\!} Z_{\text {gs}_{i}}$:

(8) $$  \text{p-value}_{\text{gs}_{j}} = 1-\Phi\left(Z_{\text{gs}_{j}}\right)  $$

### SGSE evaluation

#### Benchmark cluster-based gene set testing method

To support comparative evaluation of the SGSE method, we implemented a cluster-based gene set testing method that is representative of a large number of existing cluster and network-based gene set testing methods. Our benchmark cluster-based method computes the statistical significance of the association between gene sets and a data set as follows:

1. Cluster the *p* genomic variables in $\tilde {\mathbf {X}}$ using k-means clustering with the Hartigan and Wong algorithm [42], 5 restarts and k set according to the global maximum of the gap statistic [19] as computed using the *clusGap()* function in the *cluster* R package [43] with the number of bootstrap resamples defaulting to 100.
2. Compute the statistical significance of the association between each of the *f* gene sets defined in **A** and the k-means clustering using Pearson’s *χ* ^2^ test of independence on a 2×*k* contingency table whose first row holds the counts of gene set members in each of the k clusters and whose second row holds the total size of each of the k clusters.

#### Evaluation using simulated gene sets and simulated data

To explore the type I and type II error rates for the SGSE method and benchmark cluster-based method, a set of simulation studies were performed. In each simulation study, the SGSE method, using both choices for PC weights, and the cluster-based method were used to compute the statistical association between 10 disjoint gene sets, each of size 20, and the spectra of 1000 simulated gene expression datasets each comprised by 50 independent observations of a 200 dimension random vector simulated according to a multivariate normal distribution, ∼MVN(***0***,***Σ***). The structure of ***Σ*** varied between the simulation studies as follows:

1. Test of type I error rate: ***Σ***=**I**, reflecting a true *H*_0_.
2. Test of power using single factor design: In this case, ***Σ*** was generated as a single factor model with $\boldsymbol {\Sigma } = \lambda _{1} \boldsymbol {\alpha }_{1} \boldsymbol {\alpha }_{1}^{T} + \lambda _{d} \boldsymbol {I}$, where *λ*_1_= 4, *λ*_*d*_= 1 and ***α***_1_ is a 200-dimensional vector with all elements equal to 0 except for the first 20 which were set to $\sqrt {.05}$. This population covariance design represents a true association between the first gene set and the first PC.
3. Test of power using two factor design: In this case, ***Σ*** was generated as a two-factor model with $\boldsymbol {\Sigma } = \lambda _{1} \boldsymbol {\alpha }_{1} \boldsymbol {\alpha }_{1}^{T} + \lambda _{2} \boldsymbol {\alpha }_{2} \boldsymbol {\alpha }_{2}T + \lambda _{d} \boldsymbol {I}$, where *λ*_1_= 4, *λ*_2_= 3, *λ*_*d*_= 1, ***α***_1_ is a 200-dimensional vector with all elements equal to 0 except for the first 10 which were set to $\sqrt {.1}$ and ***α***_2_ is a 200-dimensional vector with all elements equal to 0 except for the second 10 which were set to $\sqrt {.1}\;$. This population covariance design represents a true association between the first gene set and the first and second PCs with half of the gene set associated with PC 1 and half associated with PC 2.

For all three simulation studies, the SGSE method was executed on the 1000 simulated datasets using default settings for PCGSE, as specified in Section “PCGSE for SGSE”, and both weighting methods outlined in Section “Combined significance of PCGSE p-values”. For both the single factor and two-factor simulation designs, the gap statistic method failed to predict any cluster structure, i.e., only a single cluster was predicted. To overcome this issue and prevent a 0 power result for the cluster-based method, the number of clusters used with the benchmark cluster-based method was fixed at k =2 for all simulation cases.

#### Evaluation using MSigDB C2 v4.0 gene sets and Armstrong et al. leukemia gene expression data

The SGSE method and the benchmark cluster-based method were used to compute the statistical association between the MSigDB C2 v4.0 gene sets and the spectra of the leukemia gene expression data [44] used in the 2005 GSEA paper [9]. The MSigDB C2 v4.0 gene sets and collapsed leukemia gene expression data were both downloaded from the MSigDB repository. With a minimum gene set size of 15 and maximum gene set size of 200, 3,076 gene sets out of the original 4,722 were used in the analysis. The SGSE method was executed on the leukemia gene expression data using all PCs with non-zero eigenvalues, PCGSE was called with default settings as specified in Section “PCGSE for SGSE”, and both weighting methods outlined in Section “Combined significance of PCGSE p-values” were employed. The benchmark cluster-based enrichment method was executed as outlined in Section “Benchmark cluster-based gene set testing method” (k =10 was selected as optimal by the gap statistic test). The enrichment of the MSigDB C2 gene sets was also computed relative to the acute myeloid leukemia (AML) versus acute lymphoblastic leukemia (ALL) phenotype using the competitive enrichment method CAMERA [12] with default settings. To quantify how well SGSE and the benchmark cluster-based method were able to capture the known strong association between AML/ALL status and the second PC in the data (see analysis in Frost et al. [27]), the Spearman correlation coefficient was calculated between unsupervised enrichment p-values and phenotype enrichment p-values for all MSigDB C2 gene sets. For gene sets with phenotype enrichment p-values less than 0.05, contingency table statistics were also computed measuring how well SGSE and the cluster-based enrichment method were able to identify MSigDB C2 gene sets significantly associated with the AML/ALL phenotype.

#### Evaluation using Rosenwald et al. DLBCL gene expression data and MSigDB C2 v4.0 gene sets

The SGSE method and the benchmark cluster-based method were also used to compute the statistical association between the MSigDB C2 v4.0 gene sets and the spectra of the Rosenwald et al. [45] diffuse large B-cell lymphoma (DLBCL) gene expression data. The Rosenwald et al. data set consists of gene expression measurements for 240 patients with DLBCL made using the Lymphochip microarray on 7,399 genes. Microarray data and clinical covariates from the Rosenwald et al. study were both downloaded from the paper’s supplemental information web site. To support spectral and phenotype enrichment analysis, the subset of the MSigDB C2 v4.0 gene sets whose members were measured in the Rosenwald et al. data was generated by mapping each of the Lymphochip probes, via Genbank accession numbers, to Entrez gene identifiers and MSigDB C2 v4.0 gene sets. Prior to execution of SGSE and the benchmark cluster-based method, all censored subjects were removed and missing values in the Rosenwald et al. data were imputed using k-nearest neighbor imputation using the impute.knn() function from the R impute package with default settings [46]. With a minimum gene set size of 15 and maximum gene set size of 200, 3,106 gene sets out of the original 4,722 were used in the analysis. The SGSE method was executed on the DLBCL gene expression data using all PCs with non-zero eigenvalues, PCGSE was called with default settings as specified in Section “PCGSE for SGSE”, and both weighting methods outlined in Section “Combined significance of PCGSE p-values” were employed. The benchmark cluster-based enrichment method was executed as outlined in Section “Benchmark cluster-based gene set testing method” (k =10 was selected as optimal by the gap statistic test). The enrichment of the MSigDB C2 gene sets was also computed relative to the log of survival time with the competitive enrichment method CAMERA [12] using, as a gene-level test statistic, the z-transformed t-statistic associated with the estimated coefficient from a linear model between gene expression and log survival time. To quantify how well SGSE and the benchmark cluster-based method were able to capture the association between gene set expression and survival time, the Spearman correlation coefficient was computed between unsupervised enrichment p-values and phenotype enrichment p-values for all MSigDB C2 gene sets. For gene sets with phenotype enrichment p-values less than 0.05, contingency table statistics were computed measuring how well SGSE and the cluster-based enrichment method were able to identify MSigDB C2 gene sets significantly associated with log survival time.

## Results and discussion

### Simulation example

**Figure 1:**
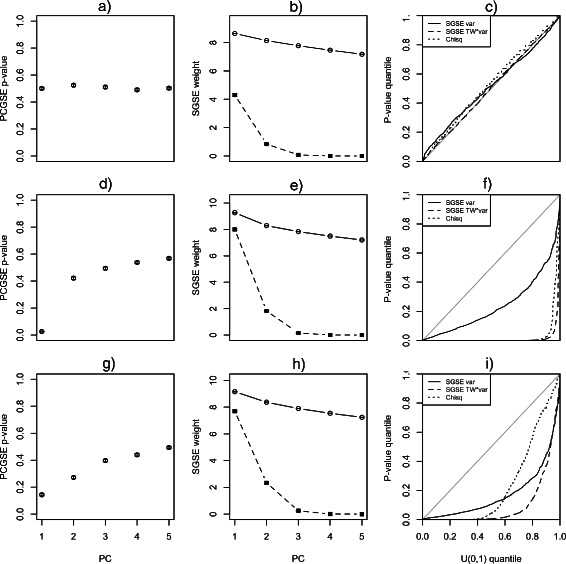
**Simulation results.** Results for the simulation studies detailed in Section “Evaluation using simulated gene sets and simulated data”. For all plots, error bars represent ±1*S* *E* for the mean value over all 1000 simulated datasets. **a)-c)** Results for the type I error simulation study based on MVN data generated with an identity population covariance matrix. This model is consistent with *H*_0_. **d)-f)** Results for the power simulation study based on MVN data generated according to a single-factor population covariance matrix. Under this model, an association exists between the first gene set and PC 1. **g)-i)** Results for the power simulation study based on MVN data generated according to a two-factor population covariance matrix. Under this model, an association exists between the first gene set and PCs 1 and 2. **a)**, **d)** and **g)** Mean p-values computed using the PCGSE method for the first simulated gene set relative to the first 5 PCs. **b)**, **e)** and **h)** Mean weights used by the SGSE method to combine the PCGSE-computed p-values for each gene set relative to the first 5 PCs. PC variance weights are shown as round points connected by a solid line. PC variance scaled by the lower-tailed p-value computed using the *Tracy-Widom* distribution for the PC variance is shown using square points connected by a dashed line. **c)**, **f)** and **i)** Quantile-quantile plot of the p-values computed using the SGSE method, with both PC variance weights (Var.) or weights defined by the PC variance scaled by the lower-tailed *Tracy-Widom* p-value of the PC variance (TW*Var.), or the benchmark method that uses a Chi-squared test between cluster membership and gene set membership (Chisq).

The three simulation studies detailed in Section “Evaluation using simulated gene sets and simulated data” were used to evaluate the type I and type II error rates for the SGSE method (using both weighting options) and the benchmark cluster-based approach. Figure 1 illustrates the results for all three simulation models.

#### Type I error rate simulation

Because the data was generated according to an identity population covariance matrix, this simulation model is consistent with the *H*_0_ of no association between any of the gene sets and any of the sample PCs. As seen in the quantile-quantile plot of unsupervised gene set enrichment p-values from the simulation study, Figure 1c), the results for all three methods are consistent with this null. At an *α*=0.05 level, the type I error rate across all 1000 simulated data sets for SGSE with variance weights was 0.02, for SGSE with Tracy-Widom scaled variance weights the type I error rate was 0.034 and for the cluster-based method the type I error rate was 0.031. These results demonstrate that all evaluated methods provide similar, and slightly conservative, control of the type I error rate.

#### Single-factor power simulation

According to the population covariance matrix used in the single-factor simulation study, only the first gene set should be significantly associated with the first PC. As seen in the Figure 1, plot d), this association is easily detected via the PCGSE method. At an *α*=0.05 level, the empirical power to detect the association between the first gene set and the spectra of the simulated data for SGSE with variance weights was 0.15, for SGSE with Tracy-Widom scaled variance weights the empirical power was 0.95 and for the cluster-based method the empirical power was 0.92. These results demonstrate that the power of the SGSE method to detect an association in the single factor case is strongly dependent on the choice of weights used to combine the PCGSE-generated p-values in the weighted Z-method, as detailed in Section “Combined significance of PCGSE p-values”. The impact of PCGSE p-value weights for the simulation example can be seen in Figure 1 plots b), e) and h). These plots show both the PC variance weights for the simulated datasets as well as weights calculated by scaling the PC variance using the lower-tailed p-value computed using the *Tracy-Widom* distribution for the PC variance. This scaled PC variance weighting results in weights being very close to the standard PC variance weights if the PC variance is highly significant according to the distribution of the principal eigenvalue of a matrix with a white Wishart distribution. As the PC variance becomes less significant, the scaling coefficient decreases lowering the effective weight for the PCGSE-computed p-value associated with that PC. Although the cluster-based method had nearly the same power as the SGSE method with Tracy-Widom scaled variance weights, it is important to note that the gap statistic only identified a single cluster in the data in this case, making the cluster-based *χ* ^2^ test for gene set enrichment meaningless if a data-driven approach is taken to determine the number of clusters.

#### Two-factor power simulation

According to the population covariance matrix used in the two-factor simulation study, the first gene set should be significantly associated with both the first and second PCs. As seen in the Figure 1, plot g), this association is detected via the PCGSE method but the signal is much less strong at the PC level than for the single-factor model. It is only by combining the measured association across all PCs that the SGSE method is able to obtain decent power in such a scenario. At an *α*=0.05 level, the empirical power to detect the association between the first gene set and the spectra of the simulated data for SGSE with variance weights was 0.31, for SGSE with Tracy-Widom scaled variance weights the empirical power was 0.71 and for the cluster-based method the empirical power was 0.52. These results again demonstrate that the power of the SGSE method to detect an unsupervised association is strongly dependent on the choice of weights used to combine the PCGSE-generated p-values in the weighted Z-method. The lower power achieved by the cluster-based method relative to the SGSE method is also noteworthy and is due, in this case, to the fact that portions of the first gene set are associated with both the first and second latent factors. This population covariance design has the effect of generating two separate correlated blocks of variables for the members of this gene set and variable clustering will therefore tend to separate them into distinct clusters, muting the ability of a *χ* ^2^ test to identify an association between cluster membership and gene set membership. Similar to the single-factor simulation, the gap statistic failed to identify more than a single cluster in the datasets simulated according to the two-factor model.

### Leukemia gene expression example

The Armstrong et al. [44] leukemia gene expression dataset and MSigDB C2 v4.0 gene sets were selected for SGSE analysis because of the known association between AML/ALL status and the spectra of the gene expression data, as illustrated in Frost et al. [27], the easy accessibility of the data and gene sets from the MSigDB repository and the common use of both the gene expression data and curated gene sets in the gene set enrichment literature (e.g., Subramanian et al. [9]).

**Figure 2:**
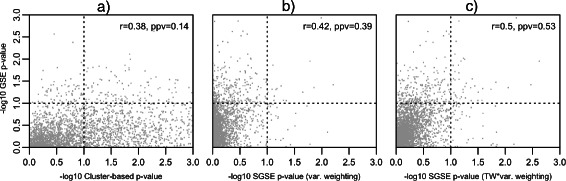
**Leukemia gene expression results.** Scatter plot showing the association between phenotype gene set enrichment p-values and unsupervised gene set enrichment p-values computed using the benchmark cluster-based method (plot **a)**) and SGSE (plots **b)** and **c)**) for the Armstrong et al. [44] leukemia gene expression data, AML/ALL phenotype, and MSigDB C2 v4.0 gene sets. Phenotype enrichment, unsupervised cluster-based enrichment and spectral gene set enrichment p-values were computed as outlined in Section “Evaluation using MSigDB C2 v4.0 gene sets and Armstrong et al. leukemia gene expression data”. Displayed in each plot is the Spearman correlation coefficient between phenotype and unsupervised gene set enrichment p-values and the positive predictive value of unsupervised gene set enrichment for identifying gene sets that are significantly enriched relative to the phenotype at an *α*=0.1 (shown by dotted lines). The results from the two different SGSE weighting methods outlined in Section “Combined significance of PCGSE p-values” are shown in plots **b)** and **c)** with **b)** plotting SGSE p-values generated using PC variance weighting and **c)** plotting SGSE p-values generated using weights defined by the PC variance scaled by the lower-tailed *Tracy-Widom* p-value for the variance.

Figure 2 shows the association between phenotype and unsupervised gene set enrichment p-values computed using both the benchmark cluster-based method and the SGSE method for the MSigDB C2 v4.0 gene sets, the AML versus ALL phenotype and the Armstrong et al. leukemia gene expression data. Although the true unsupervised enrichment status of the MSigDB C2 v4.0 gene sets relative to the variance structure of the Armstrong et al. [44] gene expression data is unknown, the phenotype enrichment results can be used as a proxy for the true unsupervised gene set enrichment based on the strong association between PC 2 and AML versus ALL status [27] as well as the recent finding by Gorlov et al. [47] that the genes with a large expression variance among cancer cases have a very high likelihood of having a known role in tumor-genesis. As indicated by the correlation between phenotype enrichment and unsupervised gene set enrichment p-values, the SGSE method was able to capture a greater proportion of the AML versus ALL enrichment signal than the benchmark cluster-based method, irrespective of the method used to weight the PC-specific gene set enrichment p-values, with the best performance obtained when PC statistical significance was used to compute the SGSE weights. The benefits of the SGSE method relative to cluster-based enrichment are most clearly visible when considering identification of AML/ALL-associated gene sets via unsupervised enrichment using a phenotype enrichment threshold of *α*=0.1. In this case, anti-conservative nature of the *χ* ^2^ test used in the cluster-based method leads to a high type I error rate and a very low positive predictive value (PPV) of 0.14 as displayed in plot **(a)**, whereas the SGSE method has a PPV 0.39 when using PC variance weights as displayed in plot **(b)** and a PPV of 0.53 when using as weights the PC variance scaled by the lower-tailed *Tracy-Widom* p-value for the variance as shown in plot **(c)**.

The *χ* ^2^ test is anti-conservative in this case because it assumes the contingency table is populated via random sampling from a common distribution. In this case, however, the contingency table is populated using genomic variables whose values are not in fact independent. Thus, the *χ* ^2^ test is using a grossly inflated sample size. Similar issues plague other uses of gene sampling in gene set testing, see Goeman et al. [30] for a discussion. Additional file 1 contains the top ten gene sets returned by each of these methods for the Armstrong et al. gene expression data. These lists clearly highlight the performance difference between SGSE and the cluster-based *χ* ^2^ test. While SGSE returns a set of cancer related gene sets in the top ten results, the *χ* ^2^ test returns gene sets without a clear cancer relationship and with very extreme p-values.

SGSE analysis of the MSigDB C2 v4.0 gene sets and Armstrong et al. [44] leukemia gene expression data illustrates the biological motivation for spectral gene set enrichment, shows the clear superiority of the SGSE approach relative to standard cluster-based gene set tests and demonstrates the importance of PC-specific p-value weights that take into account the statistical significance of each PC.

### DLBCL gene expression example

The Rosenwald et al. [45] DLBCL gene expression dataset is another good example of a clear association between the variance structure of gene expression data and an interesting clinical phenotype, in this case log survival time. Similar to the Armstrong et al. leukemia gene expression data, the Rosenwald et al. DBLCL gene expression data is easily accessible and has been widely reanalyzed in the genomics literature, factors that will support interpretation and replication of the reported SGSE results by other researchers.

**Figure 3:**
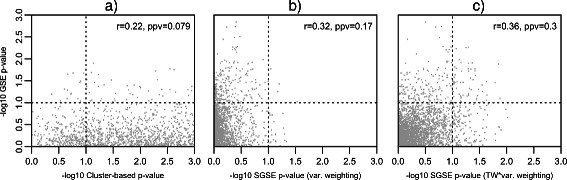
**DLBCL gene expression results.** Scatter plot showing the association between phenotype gene set enrichment p-values and unsupervised gene set enrichment p-values computed using the benchmark cluster-based method (plot **a)**) and SGSE (plots **b)** and **c)**) for the Rosenwald et al. [45] DLBCL gene expression data, log survival time phenotype, and MSigDB C2 v4.0 gene sets. Phenotype enrichment, unsupervised cluster-based enrichment and spectral gene set enrichment p-values were computed as outlined in Section “Evaluation using Rosenwald et al. DLBCL gene expression data and MSigDB C2 v4.0 gene sets”. Displayed in each plot is the Spearman correlation coefficient between phenotype and unsupervised gene set enrichment p-values and the positive predictive value of unsupervised gene set enrichment for identifying gene sets that are significantly enriched relative to the phenotype at an *α*=0.1 (shown by dotted lines). The results from the two different SGSE weighting methods outlined in Section “Combined significance of PCGSE p-values” are shown in plots **b)** and **c)** with **b)** plotting SGSE p-values generated using PC variance weighting and **c)** plotting SGSE p-values generated using weights defined by the PC variance scaled by the lower-tailed *Tracy-Widom* p-value for the variance.

Figure 3 shows the association between phenotype and unsupervised gene set enrichment p-values for the MSigDB C2 v4.0 gene sets, log survival time and the spectra of the Rosenwald et al. DLBCL gene expression data. Although the true enrichment status of the MSigDB C2 v4.0 gene sets relative to the variance structure of the Rosenwald et al. gene expression data is unknown, the phenotype enrichment results can again be used as a proxy for the true spectral gene set enrichment based on association between expression variance and cancer-related genes [47]. Although the association between SGSE and cluster-based p-values and phenotype p-values was lower for the Rosenwald et al. DLBCL gene expression data than for the Armstrong et al. leukemia gene expression data, the SGSE method was still able to capture an appreciably greater proportion of the survival time enrichment signal as compared to the benchmark cluster-based method, irrespective of the method used to weight the PC-specific gene set enrichment p-values. Similar to the findings for the leukemia gene expression data, incorporating the PC statistical significance in the SGSE weights improved the Spearman correlation between phenotype enrichment p-values and SGSE p-values for the Rosenwald et al. data. The superior performance of the SGSE method relative to the benchmark cluster-based method was again most apparent when considering identification of survival time-associated gene sets via unsupervised enrichment using just a phenotype enrichment threshold of *α*=0.1. In this case, the choice of SGSE weighting method also had a significant impact with a positive predictive value (PPV) of 0.079 for cluster-based enrichment as displayed in plot (a), a PPV of 0.17 for SGSE when using PC variance weights as displayed in plot **(b)** and a PPV of 0.3 when using as weights the PC variance scaled by the lower-tailed *Tracy-Widom* p-value for the variance as shown in plot **(c)**.

Additional file 1 contains the top ten gene sets returned by each of these methods for the Rosenwald et al. gene expression data. Similar to the top gene set lists for the leukemia gene expression data set, these lists highlight the anti-conservative nature of the *χ* ^2^ test on this dataset. The fact that the SGSE method with Tracy-Widom scaled variance weights was the only method to includes a gene set directly related to DLBCL in the top ten lends further qualitative support to the efficacy of this approach.

## Conclusions

Almost universally, gene set testing is performed in a supervised context to measure the association between functional groups of genes and a clinical phenotype. Many important examples exist, however, where a gene set-based interpretation of genomic data is desired in the absence of a phenotype variable. Although techniques have been developed for unsupervised gene set testing, they predominantly compute enrichment relative to a categorical variable defined by disjoint clusters of the genomic variables. Because such cluster-based methods often use anti-conservative contingency table-based tests and have performance that is strongly dependent on the clustering algorithm and number of clusters, they are more useful for clustering evaluation than for gene set-based interpretation of genomic data. To address the lack of effective statistical methods for unsupervised competitive gene set testing, we have developed spectral gene set enrichment (SGSE), available in the PCGSE R package from CRAN. The SGSE method first computes the statistical association between gene sets and principal components (PCs) using our principal component gene set enrichment (PCGSE) method. The overall statistical association between each gene set and the spectral structure of the data is then computed by combining the PC-level p-values using the weighted Z-method with weights set to the PC variance scaled by lower-tailed p-values from the Tracy-Widom distribution of the eigenvalue associated with each PC. On both simulated gene sets with simulated data and on curated gene sets with real gene expression data, the SGSE method has been shown to provide superior estimates of unsupervised gene set enrichment relative to standard cluster-based approaches.

## Availability of supporting data

The MSigDB C2 v4.0 gene sets can be downloaded from http://www.broadinstitute.org/gsea/msigdb/collections.jsp. The Armstrong et al. [44] leukemia gene expression data can be downloaded from http://www.broadinstitute.org/gsea/datasets.jsp. The Rosenwald et al. [45] DLBCL gene expression data can be downloaded from http://llmpp.nih.gov/DLBCL/. An implementation of the SGSE algorithm is available in the PCGSE R package (version ≥0.2, http://cran.r-project.org/web/packages/PCGSE/index.html). Due to the dependency on the Bioconductor package safe, it is recommended that PCGSE be installed using the biocLite() function. At the R prompt, enter:



## Additional file

Additional file 1
**Supplemental results for leukemia and DLBCL gene expression datasets.** Contains tables listing the top ten most significantly enriched MSigDB C2 v4.0 gene sets returned by SGSE and the cluster-based *χ* ^2^ test on the leukemia and DLBCL gene expression datasets.
