# Supplementary material for: Spectral gene set enrichment (SGSE)
Source: BMC Bioinformatics. 2015 Mar 3;16:70. doi: 10.1186/s12859-015-0490-7 (PMC4365810; doi:10.1186/s12859-015-0490-7)
Supplement: Additional file 1 — Supplemental results for leukemia and DLBCL gene expression datasets. Contains tables listing the top ten most significantly enriched MSigDB C2 v4.0 gene sets returned by SGSE and the cluster-based χ 2 test on the leukemia and DLBCL gene expression datasets. [file 12859_2015_490_MOESM1_ESM.pdf]

# Spectral gene set enrichment (SGSE)
# Additional File 1

H. Robert Frost, Zhigang Li and Jason H. Moore

## 1 Supplemental results for leukemia gene expression example

The following tables lists the top ten most significantly enriched gene sets as computed for the Armstrong et al. leukemia gene expression data set using the SGSE method (using both PC variance and Tracy-Widom scaled PC variance as weights) and the benchmark cluster-based method.

Table 1: SGSE with weight set to PC variance

| Rank | MSigDB C2 gene set | P-value (unadjusted) |
|---|---|---|
| 1 | HEDENFALK_BREAST_CANCER_BRACX_UP | 0.006139663 |
| 2 | TIEN_INTESTINE_PROBIOTICS_2HR_UP | 0.007858435 |
| 3 | TONG_INTERACT_WITH_PTTG1 | 0.010190217 |
| 4 | LEE_LIVER_CANCER_MYC_UP | 0.013862968 |
| 5 | YAMASHITA_LIVER_CANCER_WITH_EPCAM_UP | 0.016454335 |
| 6 | HILLION_HMGA1B_TARGETS | 0.016459059 |
| 7 | POMEROY_MEDULLOBLASTOMA_DESMOPLASIC_VS_CLASSIC_DN | 0.018697146 |
| 8 | ABE_INNER_EAR | 0.022428560 |
| 9 | DIRMEIER_LMP1_RESPONSE_EARLY | 0.030142783 |
| 10 | DIAZ_CHRONIC_MEYLOGENOUS_LEUKEMIA_DN | 0.031003931 |

Table 2: SGSE with weight set to PC variance scaled by Tracy-Widom p-value

| Rank | MSigDB C2 gene set | P-value (unadjusted) |
|---|---|---|
| 1 | HILLION_HMGA1B_TARGETS | 0.002367293 |
| 2 | HEDENFALK_BREAST_CANCER_BRACX_UP | 0.003418717 |
| 3 | TONG_INTERACT_WITH_PTTG1 | 0.006266318 |
| 4 | YAMASHITA_LIVER_CANCER_WITH_EPCAM_UP | 0.006980698 |
| 5 | LEE_LIVER_CANCER_MYC_UP | 0.008779404 |
| 6 | MODY_HIPPOCAMPUS_PRENATAL | 0.014196569 |
| 7 | TIEN_INTESTINE_PROBIOTICS_2HR_UP | 0.014483550 |
| 8 | DIRMEIER_LMP1_RESPONSE_LATE_DN | 0.014553060 |
| 9 | DAZARD_RESPONSE_TO_UV_NHEK_UP | 0.014920953 |
| 10 | POMEROY_MEDULLOBLASTOMA_DESMOPLASIC_VS_CLASSIC_DN | 0.015125292 |

Table 3: Cluster-based $\chi^2$ test

| Rank | MSigDB C2 gene set | P-value (unadjusted) |
|---|---|---|
| 1 | REACTOME_PEPTIDE_CHAIN_ELONGATION | 5.058823e-218 |
| 2 | KEGG_RIBOSOME | 2.164689e-211 |
| 3 | REACTOME_3_UTR_MEDIATED_TRANSLATIONAL_REGULATION | 1.234584e-185 |
| 4 | REACTOME_NONSENSE_MEDIATED_DECAY_ENHANCED_BY_THE_EXON... | 1.048964e-170 |
| 5 | REACTOME_INFLUENZA_VIRAL_RNA_TRANSCRIPTION_AND_REPLICA... | 1.141646e-169 |
| 6 | REACTOME_SRP_DEPENDENT_COTRANSLATIONAL_PROTEIN_TARGET... | 8.900224e-169 |
| 7 | REACTOME_TRANSLATION | 4.206653e-145 |
| 8 | REACTOME_INFLUENZA_LIFE_CYCLE | 9.080813e-128 |
| 9 | BILANGES_SERUM_AND_RAPAMYCIN_SENSITIVE_GENES | 7.001172e-120 |
| 10 | REACTOME_METABOLISM_OF_MRNA | 7.878889e-94 |

## 2 Supplemental results for DLBCL gene expression example

The following tables lists the top ten most significantly enriched gene sets as computed for the Rosenwald et al. DLBCL gene expression data set using the SGSE method (using both PC variance and Tracy-Widom scaled PC variance as weights) and the benchmark cluster-based method.

Table 4: SGSE with weight set to PC variance

| Rank | MSigDB C2 gene set | P-value (unadjusted) |
|---|---|---|
| 1 | SENGUPTA_NASOPHARYNGEAL_CARCINOMA_WITH_LMP1_DN | 0.02960077 |
| 2 | MARTINELLI_IMMATURE_NEUTROPHIL_DN | 0.04626866 |
| 3 | MIKKELSEN_MCV6_HCP_WITH_H3K27ME3 | 0.04954478 |
| 4 | NAKAYAMA_SOFT_TISSUE_TUMORS_PCA2_DN | 0.05006887 |
| 5 | SABATES_COLORECTAL_ADENOMA_UP | 0.05227242 |
| 6 | BOQUEST_STEM_CELL_CULTURED_VS_FRESH_DN | 0.05397167 |
| 7 | TURASHVILI_BREAST_LOBULAR_CARCINOMA_VS_DUCTAL_NORMAL_DN | 0.06022983 |
| 8 | KEGG_RENIN_ANGIOTENSIN_SYSTEM | 0.06310880 |
| 9 | KIM_BIPOLAR_DISORDER_OLIGODENDROCYTE_DENSITY_CORR_DN | 0.06669102 |
| 10 | MURAKAMI_UV_RESPONSE_24HR | 0.07258131 |

Table 5: SGSE with weight set to PC variance scaled by Tracy-Widom p-value

| Rank | MSigDB C2 gene set | P-value (unadjusted) |
|---|---|---|
| 1 | KEGG_ECM_RECEPTOR_INTERACTION | 0.009658082 |
| 2 | PID_INTEGRIN1_PATHWAY | 0.010380034 |
| 3 | TURASHVILI_BREAST_DUCTAL_CARCINOMA_VS_DUCTAL_NORMAL_DN | 0.012747625 |
| 4 | VECCHI_GASTRIC_CANCER_ADVANCED_VS_EARLY_UP | 0.013251721 |
| 5 | SHIPP_DLBCL_VS_FOLLICULAR_LYMPHOMA_DN | 0.013551958 |
| 6 | JEON_SMAD6_TARGETS_UP | 0.013926605 |
| 7 | BOQUEST_STEM_CELL_UP | 0.014167598 |
| 8 | MIKKELSEN_MCV6_HCP_WITH_H3K27ME3 | 0.016180259 |
| 9 | LI_WILMS_TUMOR_VS_FETAL_KIDNEY_2_DN | 0.016260646 |
| 10 | ONDER_CDH1_TARGETS_2_UP | 0.016401605 |

Table 6: Cluster-based $\chi^2$ test

| Rank | MSigDB C2 gene set | P-value (unadjusted) |
|---|---|---|
| 1 | SOTIRIOU_BREAST_CANCER_GRADE_1_VS_3_UP | 1.294159e-112 |
| 2 | ROSTY_CERVICAL_CANCER_PROLIFERATION_CLUSTER | 5.652931e-106 |
| 3 | CROMER_TUMORIGENESIS_UP | 7.386337e-103 |
| 4 | GRAHAM_CML_DIVIDING_VS_NORMAL_QUIESCENT_UP | 4.668787e-83 |
| 5 | WHITEFORD_PEDIATRIC_CANCER_MARKERS | 2.026694e-82 |
| 6 | ZHOU_CELL_CYCLE_GENES_IN_IR_RESPONSE_24HR | 1.250324e-81 |
| 7 | WINNEPENNINCKX_MELANOMA_METASTASIS_UP | 1.898323e-78 |
| 8 | BENPORATH_PROLIFERATION | 4.228859e-76 |
| 9 | CROONQUIST_IL6_DEPRIVATION_DN | 1.225128e-75 |
| 10 | BROWNE_INTERFERON_RESPONSIVE_GENES | 1.810688e-75 |
